# Hereditary thrombophilia and thrombosis of tunneled hemodialysis catheters: A single center study

**DOI:** 10.34172/jcvtr.2021.06

**Published:** 2021-01-19

**Authors:** Farzad Kakaei, Saba Mirabolfathi, Negin Yavari, Mohammad Reza Ardalan, Mehrdad Mozafar, Sina Zarrintan

**Affiliations:** ^1^Department of General Surgery, Imam Reza Hospital, Tabriz University of Medical Sciences, Tabriz, Iran; ^2^Section of Organ Transplantation, Tabriz University of Medical Sciences, Tabriz, Iran; ^3^Faculty of Medicine, Tabriz University of Medical Sciences, Tabriz, Iran; ^4^Research Department, Tehran Heart Center, Tehran University of Medical Sciences, Tehran, Iran; ^5^Division of Nephrology, Department of Internal Medicine, Imam Reza Hospital, Tabriz University of Medical Sciences, Tabriz, Iran; ^6^Faculty of Medicine, Tehran University of Medical Sciences, Tehran, Iran; ^7^Cardiovascular Research Center, Tabriz University of Medical Sciences, Tabriz, Iran

**Keywords:** Thrombophilia, Hemodialysis, Thrombosis, Tunneled Hemodialysis Catheter

## Abstract

***Introduction:*** Vascular access thrombosis increases the risk of mortality and morbidity in end-stage renal disease (ESRD) patients on hemodialysis (HD). This study aimed to evaluate hereditary thrombophilia factors in HD patients and its association with tunneled cuffed catheters’ thrombosis.

***Methods:*** In this cross-sectional study, 60 consecutive patients with ESRD on HD with tunneled cuffed catheters were selected. Inherited thrombophilia factors (Anti-thrombin III, Protein C, Protein S, and Factor V Leiden) were measured and the patients were followed for 3 months to evaluate the incidence of catheter-related thrombosis. The association between these factors and catheter thrombosis was assessed.

***Results:*** The mean age of patients was 60.30 ± 8.69 years. Forty-seven patients (78.30%) were female and thirteen patients (21.70%) were male. The most common cause of ESRD was diabetes mellitus (41.67%). The most catheter site was the right internal jugular vein (55%). There were 22 (36.67%) and 8 (13.33%) cases of thrombosis and mortality, respectively. The association between hereditary thrombophilia factors and catheter thrombosis was not statistically significant (*P* > 0.05).

***Conclusion:*** In this small group of our patients, the frequency of hereditary thrombophilia was not significantly different between those with and without thrombosis of tunneled HD catheter.

## Introduction

Hemodialysis (HD) is still a common method of renal replacement therapy (RRT) in patients with end-stage renal disease (ESRD).^1,2^ Although the best option for hemodialysis is creation of a native arteriovenous fistula (AVF), tunneled cuffed HD catheters are still used in a considerable number of patients.^3-5^ However, complications of catheters are high and they contribute to potential morbidities and mortalities in some patients with ESRD.^1,6,7^ Catheter-related complications are more common during the first 90 days of placement.^5,8^ The activation of coagulation cascade has been studied to be extensively related to thrombosis. Thrombophilia plays an important role in thrombosis in contribution to other factors. Deficiency of anticoagulation proteins, including anti-thrombin III, protein C and S and factor V Leiden is associated with inherited thrombophilia which enhances the hypercoagulability state in patients on HD.^9-11^ Investigating the role of inherited thrombophilia factors may help decrease the risk of catheter failure due to thrombosis in such patients. There is a paucity of data on the role of hereditary thrombophilia for thrombosis of a tunneled HD catheter in ESRD patients. In this study, we aimed to evaluate the role of hereditary thrombophilia factors associated with permanent tunneled catheter thrombosis among patients undergoing HD.

## Materials and Methods

### Study population

**Figure 1:**
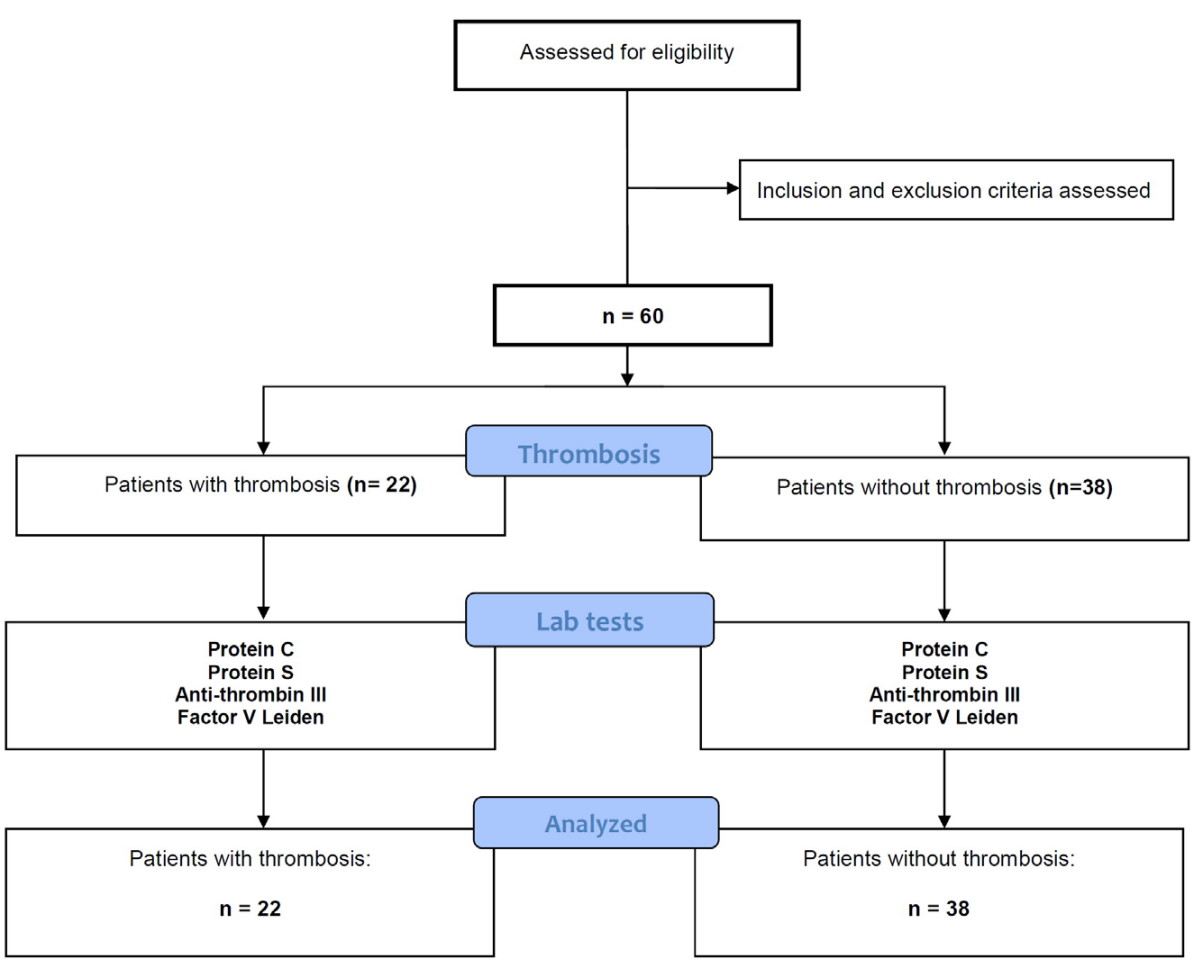
Flow chart of the study patients

The present cross-sectional study included 60 consecutive patients with ESRD who were candidates of cuffed tunneled HD catheter placement at Imam Reza Hospital of Tabriz University of Medical Sciences, Tabriz, Iran. The study was conducted from March 2012 to November 2012 and the cases were selected according to the inclusion and exclusion criteria. The study protocol was approved by the ethics committee of Tabriz University of Medical Sciences under the number 91/1-1/2 and ethical code of 5/4/6862. Figure 1 illustrates the flow chart of the study participants.

### Inclusion and exclusion criteria

Patients who were on chronic HD, aged between 16 and 80 years old, and had a history of unsuccessful placement of arteriovenous fistula or arteriovenous graft were selected for the study. Tunneled HD catheters were placed in the jugular or subclavian veins without any technical problems and the patients underwent 3 times of HD for four hours without any complications. Of the total patients, only 60 patients met our criteria to enter the study. Patients who received oral anticoagulation or contraceptives, had acute or chronic hepatitis, systematic lupus erythematosus or other immunological or rheumatologic diseases, malignancy, diabetes, exit site infection, technical errors in the placement of the catheter, replacement of the catheter, history of deep vein thrombosis (DVT), recent surgery and immobilization were excluded from the study.

### Blood sampling and study variables

Before the beginning of HD, blood samples were obtained from peripheral veins to measure routine blood cell counts and coagulation factors, including prothrombin time (PT), international normalized ratio (INR), protein C and S levels, anti-thrombin III, lupus anti-coagulant and factor V Leiden. The patients were followed for three months.

Patients’ characteristics were consisted of age, gender, body mass index (BMI), presence of hypertension before hemodialysis, cause of renal failure, anatomical location of catheter and time of exchanging the catheter. We used cuffed tunneled catheters with tip to cuff length of 19cm, 23cm and 28cm depending on the patients’ anatomy and the used central vein. All the catheters were from a same company. All the catheters were placed by Seldinger’s technique and tunneled subcutaneously. The location and function of catheters were confirmed by chest X-ray and successful hemodialysis within 24 hours of placement, respectively. All the patients received an appropriate dosage of heparin (100-150 UI/kg) before the beginning of HD. In the end, all the catheters were irrigated with normal saline and filled with heparin. Patients were followed by phone calls and office visits if the catheters failed to function at routine HD sessions. Diagnosis of thrombosis was initially made by documentation of inability to perform HD or absence of sufficient flow, followed by ultrasound confirmation. Thrombophilia markers were assessed in all the patients. Thrombophilia was confirmed if the patients had anti-thrombin activity <75%, protein C and protein S activity <70%, positive lupus anticoagulant, and factor V Leiden.

### Statistical analysis

Statistical comparison between quantitative variables was conducted by independent T-test. Chi-square test and Fisher’s exact test were used to identify qualitative differences between groups. A p-value less than 0.05 was considered to be statistically significant. All statistical analyses were conducted using IBM SPSS Statistics for Windows, version 24.0 (Armonk, NY: IBM Corp.).

## Results

A total of 60 patients with ESRD were investigated. The mean age of patients was 60.30 ± 8.69 years. Forty-seven (78.30%) patients were female and thirteen (21.70%) patients were male. The mean systolic blood pressure was 112.50 ± 2.80 mm Hg. The most common causes of ESRD were diabetes (n= 25, 41.67%) and hypertension (n= 19, 31.67%). Fourteen patients (23.33%) had unknown causes and two patients (3.33%) had chemotherapy-induced ESRD.

The mean period of ESRD was 16.56 ± 15.92 months. The most common site of catheter placement was right internal jugular vein (n= 33, 55%). In addition, eleven (18.33%) catheters were placed through left internal jugular vein. Right and left subclavian accesses were used in ten (16.67%) and six (10%) patients, respectively.

Catheter thrombosis and perioperative mortality occurred in 22 (36.67%) and 8 (13.33%) cases, respectively. Laboratory findings are shown in Table 1. In addition, hereditary thrombophilia factors are presented in Table 2. PT and INR were not significantly different between the groups (*P* > 0.05). Also, mean values of protein C, protein S and anti-thrombin III were not significantly different between the two groups (*P* > 0.05; independent sample t-test). We found no significant differences in the numbers of patients with lower limit protein C, protein S, anti-thrombin III deficiency and positive factor V Leiden between the two groups (*P* > 0.05; chi-square test). All the cases were negative for lupus anticoagulant.

**Table 1:** Laboratory findings of the study population

**Laboratory variables**	**Mean**	SD***	**Maximum**	**Minimum**
PT*	12.56	1.08	11	16
INR**	1.04	0.07	1	1.30
Protein C	77.60	26.64	37	151
Protein S	92.91	34.59	37	160
Anti-thrombin III	91.87	21.02	31	128

Abbreviations: PT, prothrombin time ;INR, international normalized ratio Prothrombin time; **International normalization ratio; ***Standard Deviation

**Table 2:** Hereditary thrombophilia factors in patients with or without thrombosis

	**With thrombosis** ^a^ **(n= 22 patients)**	**Without thrombosis** ^a^ **(n= 38 patients)**	***P*** ** value** ^b^
PT ^c^	12.41± 0.91	12.63 ± 1.18	0.44
INR ^d^	1.04 ± 0.06	1.04 ± 0.08	0.93
Mean Protein C^e^ Normal Decreased	73.36 ± 24.10 14 (63.60%) 8 (36.40%)	80.06 ± 28.01 25 (65.80%) 13 (34.20%)	0.35 0.86
Mean Protein S^e^ Normal Decreased	94.22 ± 31.54 18 (81.80%) 4 (18.20%)	92.15 ± 36.63 28 (73.70%) 10 (26.30%)	0.82 0.47
Mean Anti-thrombin III^f^ Normal Decreased	90.34 ± 31.54 19 (86.4%) 3 (13.60%)	92.76 ± 23.90 32 (84.20%) 6 (15.80%)	0.67 0.82
Factor V Leiden Positive Negative	21 (95.50%) 1 (4.50%)	37 (97.40%) 1 (2.60%)	0.90

Abbreviations: PT, prothrombin time ;INR, international normalized ratio ^a^The comparison between two groups was conducted by independent sample *t* test for quantitative measures and with chi-square test for qualitative measures. ^b^ *P* values more than 0.05 indicate insignificant differences ^c^Prothrombin time ^d^International normalized ratio ^e^The measures are presented by laboratory reports of percentages of activity

## Discussion

The rapid growth of ESRD is accompanied by increased use of permanent HD vascular accesses. In addition, because most of the HD patients who use catheters for dialysis have history of failed arteriovenous fistulas and grafts, patency and function of catheter are of potential importance. ESRD patients who are on HD by cuffed tunneled catheter require long-term function of the catheter. However, thrombosis, infection and malfunction predispose the patients to potential morbidities and mortalities. Catheter-related thrombosis itself are of the most important complications of tunneled HD catheter, leading to access dysfunction, decreased quality of life and increased rate of mortality.^12,13^

Thrombophilia predisposes patients with ESRD to venous and catheter thrombosis. It may be initiated by both genetic and acquired factors. Genetic factors include genetic mutations of hemostasis factors which potentially increase the risk of thrombosis. Such genetic factors are not always associated with thrombosis and are age-dependent. Hereditary thrombophilia includes deficiencies of anti-thrombin III, protein C and S, and mutation in factor V Leiden genes.^10^ Although the last one is responsible for more than 50% of cases, it is associated with mild thrombophilia.^10,14^ Moreover, mutations in prothrombin gene is associated with lower risk, protein C and S deficiencies with moderate risk and anti-thrombin Ⅲ deficiencies with higher risk of thromboembolic events.^14^

Knoll et al. evaluated the association between thrombophilia and arteriovenous fistula or graft thrombosis in 419 hemodialysis patients. They observed that 55% of patients with vascular access thrombosis had at least one of thrombophilia factors.^7^ In a study by Rios et al., factors involved in the occurrence of thrombosis in the 195 HD patients were identified. They observed that Factor 5 Leiden, and blood groups did not have correlation with thrombosis, but prothrombin gene mutation was significantly higher in patients with acute thrombosis.^2^ Ataç et al. investigated the role of genetic mutation in vascular thrombosis in the HD patients waiting for renal transplant. They found that hereditary thrombophilia was common cause of thrombosis but it was influenced by various environmental factors, such as hormone ingestion, immobilization, oral contraceptive pills and surgery. Moreover, PT 20210 mutation put patients at significantly higher risk of developing thrombosis.^11^ Moreover, Chen et al. found that the incidence of protein S, protein C, anti-thrombin Ⅲ deficiency and elevated anti-cardiolipin antibody were not significantly higher in the group of patients who experienced early access occlusion after angioplasty than those without occlusion for at least 30 days. However, all together, they were significantly more prevalent in the case group, suggesting the role of hereditary thrombophilia factors in early vascular access thrombosis.^15^ In a study by Akman et al., access thrombosis was only related to factor V Leiden mutation.^16^

A meta-analysis reviewed 16 cohort study and showed that elevated factor Ⅷ, protein C deficiency and factor Ⅴ leiden mutation were related to central venous catheter-associated deep vein thrombosis (CADVT) in children.^17^ Also, another meta-analysis on patients with cancer investigated the association between hereditary thrombophilia abnormalities and central venous catheter thrombosis. They showed that the presence of factor V Leiden and prothrombin G20210A mutation were related to the central venous catheter thrombosis.^18^ Decousus et al. noted that antiphospholipid antibodies and acquired resistance to activated protein C predispose the cancer patients to thrombosis. Presence of all the hereditary thrombophilia factors were not associated with increased risk of thrombosis.^19^ In addition, another study by Boersma et al. was performed on patients with cancer. They evaluated the risk of central venous catheter thrombosis with inherited thrombophilia factors. They found that only the patients with mutation of factor V Leiden would develop thrombosis.^8^ However, we found that protein C, S, and anti-thrombin III deficiencies and factor V Leiden mutation were not associated with catheter thrombosis in HD patients. The major difference between our study and the previous studies is that they conducted their study on patients with preexisting condition of cancer, which could have affected the result due to potential acquired thrombophilia caused by cancer.

Nampoory et al. performed a study on HD patients who had hypercoagulable state. Patients who experienced venous thrombosis compared to those without thrombosis had significantly abnormal parameters of lupus anticoagulant, protein C, protein S and activated protein C resistance, though their hyper-coagulation was corrected after renal transplantation.^20^ In the present study there was no significant association between the incidence of catheter thrombosis and hereditary thrombophilia factors.

Small sample size was the main limitation of our study. Investigating the tunneled hemodialysis catheter thrombosis for possible association with hereditary thrombophilia was the major advantage of our study, while previous studies were mostly evaluated other vascular accesses for this purpose. Future studies with larger sample size are necessary to further elucidate this relationship. Likewise, catheter brand may also affect the without-thrombosis duration.Thus, a more comprehensive study with the evaluation of different types of catheters together with thrombophilia factors would assess the association of these factors and catheter thrombosis more precisely. In addition, a prospective study with precise stratification of background characteristics would potentially strengthen the likelihood of this association.

## Conclusion

In conclusion, in this small group of our patients, the frequency of hereditary thrombophilia was not significantly different between those with and without thrombosis of tunneled HD catheter.

## Acknowledgements

The authors acknowledge all general surgery residents of Tabriz University of Medical Sciences, Tabriz, Iran who were involved in care of the presented patients. The authors also thank Dr. Annis Shahnaee for her useful comments during the preparation of this article.

## Competing interest

The authors do not have any conflicts of interest.

## Ethical approval

The study protocol was approved by the ethics committee of Tabriz University of Medical Sciences under the number 91/1-1/2 and ethical code of 5/4/6862.

## Funding

Research Deputy, Faculty of Medicine, Tabriz University of Medical Sciences, Tabriz, Iran.
